# Increased patellar bone tracer uptake in preoperative SPECT/CT before medial opening high tibial osteotomy correlates with inferior clinical outcome

**DOI:** 10.1007/s00167-021-06717-2

**Published:** 2021-09-05

**Authors:** B. L. Schelker, C. S. Moret, O. Dogan, F. Amsler, H. Rasch, R. W. Hügli, M. T. Hirschmann

**Affiliations:** 1 Department of Orthopaedic Surgery and Traumatology, Kantonsspital Baselland (Bruderholz, Liestal, Laufen), 4101 Bruderholz, Switzerland; 2 University of Basel, Basel, Switzerland; 3 Amsler Consulting, Basel, Switzerland; 4 Institute of Radiology and Nuclear Medicine, Kantonsspital Baselland (Bruderholz, Liestal, Laufen), 4101 Bruderholz, Switzerland

**Keywords:** Knee, Outcome, High tibial osteotomy, Medial compartment osteoarthritis, SPECT/CT, Alignment

## Abstract

**Purpose:**

The purpose of this study was to investigate whether specific bone tracer uptake (BTU) patterns on preoperative SPECT/CT could predict which patients with varus alignment and medial overload would particularly benefit from medial opening-wedge high tibial osteotomy (MOWHTO). It was the hypothesis that an increased preoperative BTU relative to the reference BTU of the femur on SPECT/CT in the lateral and patellar compartments of the knee are predictive factors for inferior clinical outcome and that the clinical outcome correlates with the extent of alignment correction.

**Methods:**

Twenty-three knees from 22 patients who underwent MOWHTO for medial compartment overload were investigated preoperatively using Tc-99m-SPECT/CT. BTU was quantified and localised to specific joint areas according to a previously validated scheme. Pre- and postoperative mechanical alignment was measured. Clinical outcome was assessed at a median of 24 months (range 11–30) after MOWHTO by collecting the WOMAC score.

**Results:**

Significant correlations between BTU in the patellar area and the total WOMAC score and its subcategories pain and stiffness were found. Thus, BTU in the 1sPat area (superior lateral patellar compartment) correlated with total WOMAC (rho = 0.43, *p* = 0.04), pain subcategory (rho = 0.43, *p* = 0.04), and stiffness subcategory (rho = 0.59, *p* = 0.003). No significant correlations were found between alignment correction, age, gender and WOMAC.

**Conclusion:**

This study highlights the role of preoperative SPECT in modern knee surgery to obtain information about the loading pattern on different compartments of the knee. Despite the limited number of participants, the present study shows that a preoperative SPECT/CT scan can help the treating surgeons to identify patients who may be at risk of inferior clinical outcome if an MOWHTO is considered, as an elevated BTU in the patellar region on preoperative SPECT/CT appears to be a potential risk factor for postoperative pain and stiffness.

**Level of evidence:**

Level III.

## Introduction

One cause of medial compartmental osteoarthritis (OA) is varus alignment of the lower limb. In a varus knee, the weight-bearing axis runs medially through the knee and results in the majority of the load being transferred to the medial compartment [7, 22]. To shift the load from the medial to the lateral compartment of the knee and thus relieve pain and delay the progression of OA, a valgus high tibial osteotomy might be considered [9]. To date, medial opening-wedge high tibial osteotomy (MOWHTO) is the method of choice when compared with lateral closing-wedge HTO due to its less demanding technique and reduced complication rate [18, 35]. There is a considerable variation in the literature regarding the ideal postoperative mechanical alignment. The majority of authors indicate that correction should extend beyond the neutral position [16, 34]. Nevertheless, others suggest that overcorrection leads to the progression of degenerative changes in the lateral compartment of the knee [14, 23, 33]. Hirschmann et al. showed that single-photon emission computed tomography/computed tomography (SPECT/CT) can be used to visualise and quantify the loading pattern of the knee in comparison to the mechanical alignment [11]. Furthermore, Mucha et al. were able to show that the load in the medial compartment decreased in patients with varus alignment after MOWHTO. The decrease in load resulted in a decrease in bone tracer uptake (BTU) and was correlated with a reduction in pain and stiffness in patients. Conversely, a higher postoperative BTU has been shown to be associated with increased pain [26].

The primary purpose of the study was to investigate whether findings in SPECT/CT can predict which patients with varus alignment and medial overload after MOWHTO will particularly benefit from MOWHTO and which will not. It was hypothesised that an increased preoperative BTU on SPECT/CT in the lateral and patellar compartments is a predictive factor for a worse outcome and therefore patients with such finding benefit less from MOWHTO. The secondary objective was to evaluate the clinical outcome compared to the amount of correction of the mechanical alignment. It was hypothesised that the postoperative shift of the mechanical axis may lead to additional loading on the left compartment and thus cause pain and worse clinical outcome, especially in case of overcorrection.

## Materials and methods

IRB approval was obtained from the local ethics committee of Northwestern Switzerland (EKNZ, application number 2016-01349). All procedures performed were in accordance with the ethical standards of the responsible committee and with the 1964 Declaration of Helsinki and its later amendments. Informed consent was obtained from all individual participants prior to enrolment in the study.

The hospital archive was searched for patients who underwent MOWHTO for medial joint compartment overload due to mechanical varus alignment. Twenty-three knees from 22 consecutive patients were retrospectively included in this study. In all patients, a SPECT/CT of the affected joint was performed preoperatively. The mean age was 47 ± 10 years at the time of surgery. The enrolled cohort was composed of 18 males (19 knees) and 4 females (4 knees) (Table 1).

**Table 1:** Baseline characteristics

	Mean (SD)	*N* knees (%)
Age OP	46,5 (± 10.4)	
Sex		
Male		19 (82.6)
Female		4 (17.4)
Side		
Left		13 (56.5)
Right		10 (43.5)

Exclusion criteria were post-traumatic OA, open growth plates, use of corticosteroids within the last 6 months, osteonecrosis, osteochondritis dissecans, chondrocalcinosis of the meniscus, tumour diseases, Paget's disease, knee joint infection, periarticular fracture, neuropathic arthropathy, reactive arthritis, gout, as well as patients with dementia or other limitations who could not complete the clinical outcome questionnaire.

MOWHTO was performed by experienced orthopaedic surgeons using published standard surgical techniques with the Tomofix plate (Synthes, Oberdorf, Switzerland) or the Numelock plate (Stryker, Selzach, Switzerland) [17, 21, 28, 30]. Preoperative SPECT/CT was performed using a hybrid system (Symbia T16 Siemens, Erlangen, Germany) and using 99mTc-HDP 500-7000 MBq (Mallinckrodt, Wollerau, Switzerland) as radionuclide. Scintigraphic images were acquired in three phases, the perfusion phase immediately after injection, the blood pool phase 2–5 min after injection and the delayed metabolic phase 2–3 h after injection. The SPECT/CT was performed with a matrix of 128 × 128, an angle step of 32 degrees and a time per frame of 25 s. 3D reconstruction of the images was made.

**Fig. 1:**
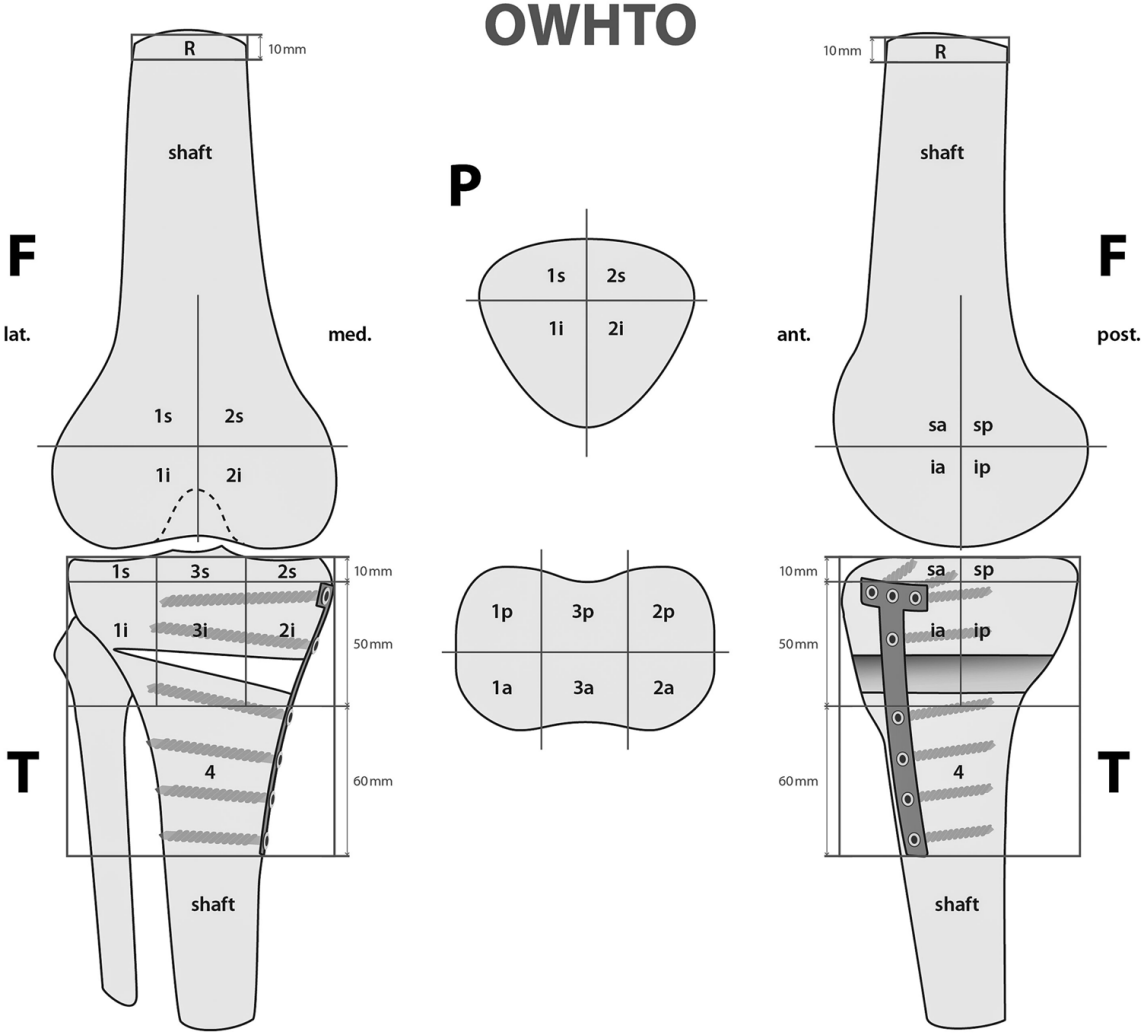
The mapping scheme used for localization of areas of increased SPECT/CT tracer uptake values (F 1⁄4 femur; T 1⁄4 tibia; P 1⁄4 patella; 1 1⁄4 lateral; 2 1⁄4 medial; 3 1⁄4 tibial intercondylar area; s 1⁄4 superior; i 1⁄4 inferior; a 1⁄4 anterior; p 1⁄4 p

**Fig. 2:**
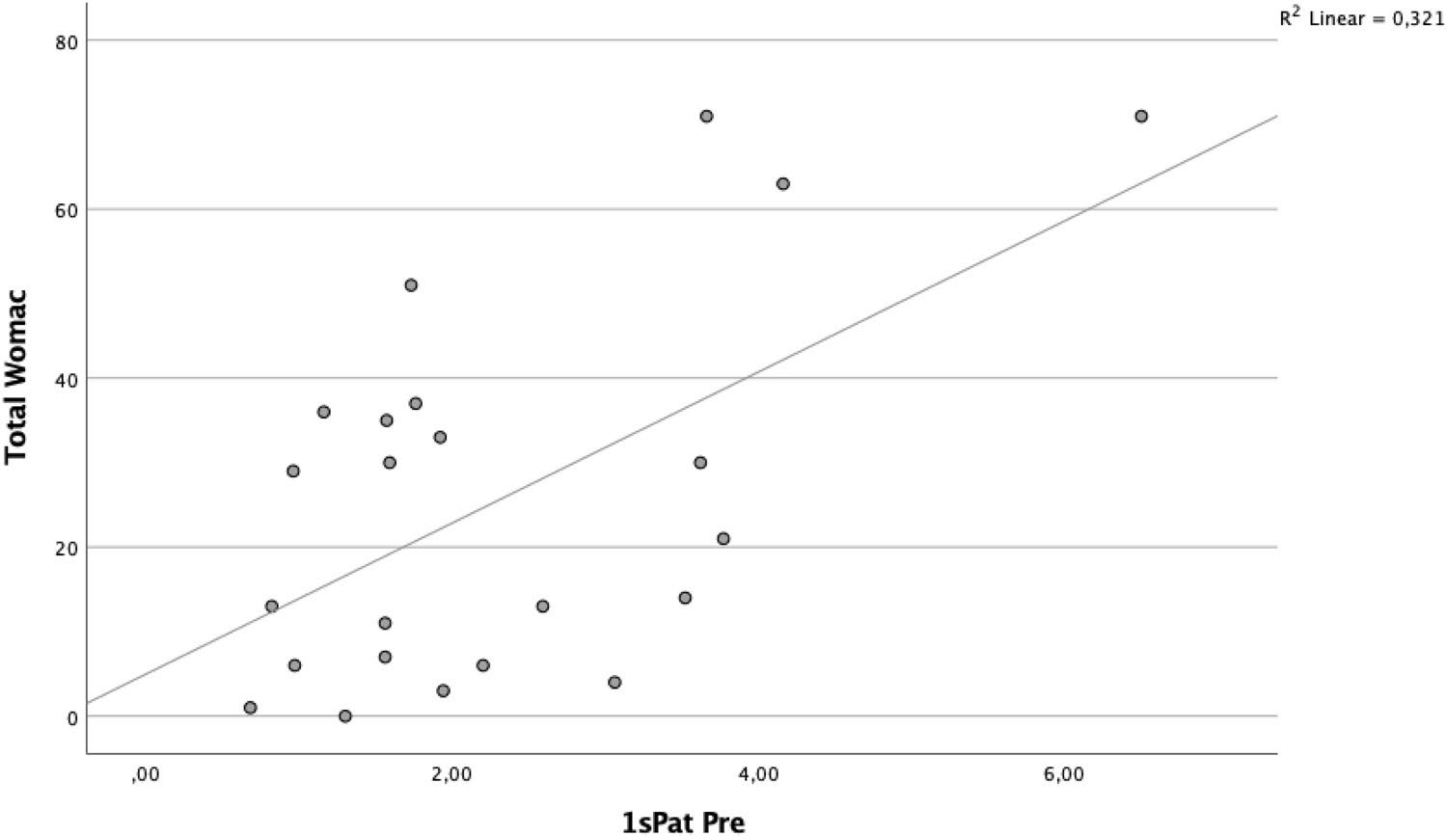
Relationship between total WOMAC score and BTU in area 1sPat

**Fig. 3:**
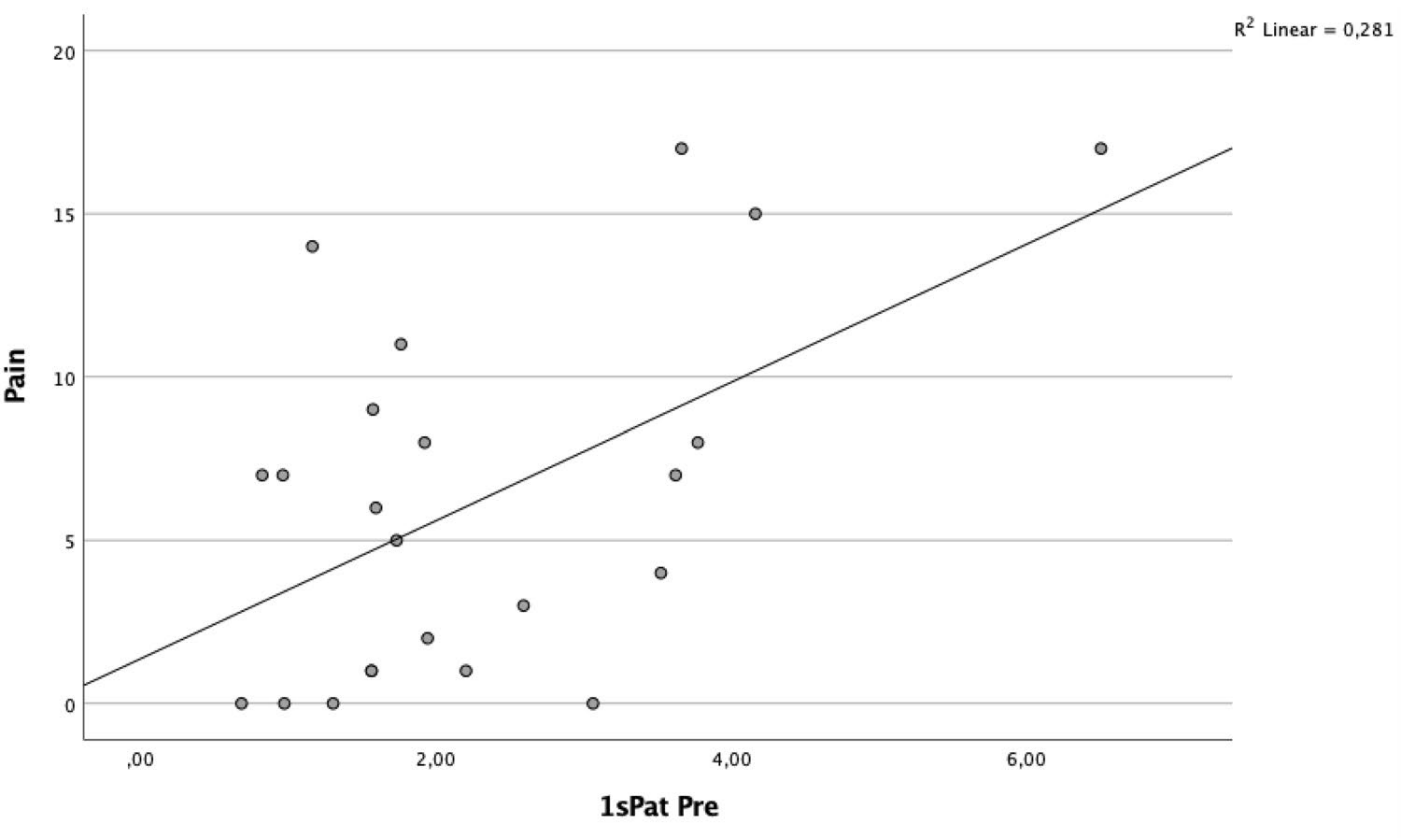
Relationship between total WOMAC pain score and BTU in area 1sPat

**Fig. 4:**
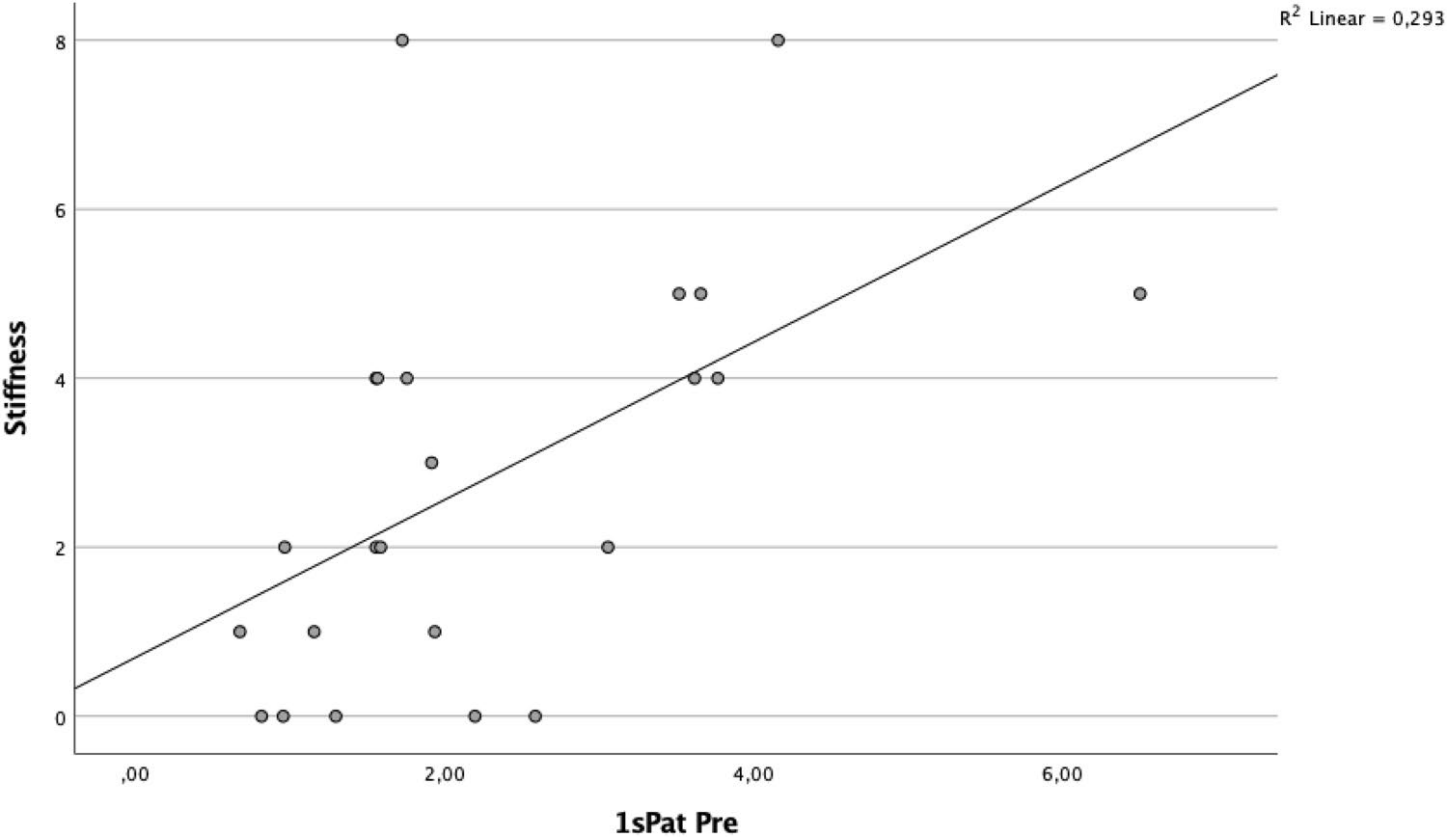
Relationship between WOMAC stiffness score and BTU in area 1sPat

The BTU was analysed using custom software (IntroSPECT, OrthoImagingSolutions Ltd., London, UK) which is able to localise the BTU according to a previously validated and standardised localisation scheme [12, 27]. The scheme defines 9 femoral, 8 patellar and 13 tibial zones to map the BTU volume (Figs. 1, 2, 3, 4). Each zone of the femur and tibia is represented with a number (1—lateral,—-medial, 3—tibial) and two small letters (a—anterior, p—posterior, i–inferior, s—superior). The patella is divided into four zones (2s—superomedial, 1s—superolateral, 2i—inferomedial and 1i—inferolateral). Values for each were recorded and presented as a numerical value (mean ± standard deviation, median and range) in relation to the reference recording of the bone shaft of the femur.

Mechanical alignment was measured as the angle between a line connecting the femoral head to the intercondylar notch and with a line connecting the centre of the talus to the tibial eminence. It was measured by SPECT/CT using the previously validated software (Orthoexpert v1.15©, OrthoImagingSolutions Ltd, London, UK) [31].

All measurements were performed twice by two independent observers at an interval of 4 weeks. Both observers were blinded to the results of the previous observations. The localisation scheme showed very high inter- and intraobserver reliability (OR) (intra-class correlation coefficient (ICC) > 0.9) for the BTU and localisation measurements. There was also a strong inter-observer agreement for the mechanical alignment measurements (an inter-OR of ICC = 0.99 and an intra-OR of ICC = 0.98).

The clinical outcome was assessed at a median of 24 months (range 11–30 months) post-surgery using the Western Ontario and McMaster Universities Osteoarthritis Index (WOMAC) questionnaire. The WOMAC score contains 24 questions and is able to assess the effects of osteoarthritis of the knee joint in terms of pain, stiffness and the daily activities of the patient [38]. The WOMAC score has been used in the past in several studies as a patient-reported outcome measure (PROM) to evaluate clinical outcome after HTO [1, 13].

### Statistical analysis

Data were analysed using SPSS 17.0 and 27.0 (SPSS, Chicago, IL). Continuous variables were described using means and standard deviations or medians and ranges. Non-parametric Spearman correlations were used to calculate associations between mechanical axis correction, preoperative BTU uptake and WOMAC score. A non-parametric test (Wilcoxon) was used to compare the BTU of preoperative SPECT/CT of specific localizations. ETA correlations were determined for correlations between sex and WOMAC score. For all analyses, a *p* value less than 0.05 was considered statistically significant. A post hoc power analysis tested that for the number of subjects given, *n* = 23, a correlation of rho ≥ 0.55 with a power of 80% can be found, considering a two-tailed *p* value less than 0.05. With a one-tailed *p* value less than 0.05, a correlation of rho ≥ 0.50 can be found with a power of 80%.

### Results

Compared to the lateral compartment, the medial compartment shows significantly higher BTU (Table 2). Details of the preoperative and postoperative mechanical axis and as well as their difference are provided in Table 3. The mean values for the WOMAC total score and the subcategories pain, stiffness and daily activities are also shown in Table 3.

**Table 2:** Comparison of BTU of medial (= 2) and lateral (= 1) compartments and Wilcoxon rank sum test

	Lateral		Medial		*p*
	Mean (SD)	Min, 25% 50% 75% max	Mean (SD)	Min, 25% 50% 75% max	
saFe	1.77 (0.64)	1.00, 1.36, 1.56, 2.05, 3.70	1.77 (0.59)	1.03, 1.31, 1.66, 2.12, 3.20	ns
spFe	2.01 (1.29)	1.24, 1.4, 1.49, 2.37, 7.44	2.10 (1.04)	0.98, 1.39, 1.74, 2.50, 4.91	ns
iaFe	2.03 (0.87)	0.88, 1.35, 1.62, 2.64, 4.28	3.30 (1.66)	1.33, 1.74, 2.95, 4.52, 7.27	0.001
ipFe	2.02 (1.26)	0.83, 1.35, 1.65, 2.19, 6.96	3.79 (1.99)	1.28, 2.10,3.58, 4.93, 9.31	0.000
saTib	1.56 (0.61)	0.92, 1.12, 1.32, 1.98, 3.34	2.86 (1.65)	1.24, 1.73, 2.47, 3.35, 8.49	0.000
spTib	1.76 (0.50)	1.13, 1.44, 1.57, 2.08, 2.97	3.41 (1.97)	1.52, 1.90, 3.06, 4.10, 10.47	0.000
iaTib	1.83 (0.68)	0.58, 1.21, 1.80, 2.21, 3.21	1.70 (1.02)	0.44, 1.15, 1.55, 1.80, 5.12	ns
ipTib	1.62 (0.44)	1.00, 1.35, 1.48, 1.82, 2.64	2.29 (1.39)	0.99, 1.44, 1.95, 2.75, 7.49	0.013

**Table 3:** Mean values and standard deviation of alignment and postoperative WOMAC score

	*N*	Mean	Median	SD	Min	Max	Percentile		
							25	50	75
MecAx Pre	23	2.73	3.00	2.86	− 2.30	7.30	0	3	5
MecAx Post	23	− 2.83	− 2.9	2.54	− 9.8	1.3	− 4.3	− 2.9	− 1
MecAx Diff	23	− 5.53	− 5.88	2.77	− 11.38	2	− 7.5	− 5.88	− 4
Pain	23	6.22	6	5.56	0	17	1	6	9
Stiffness	23	2.83	2	2.41	0	8	1	2	4
Daily activities	23	17.43	19	16.93	0	57	4	19	22
Total WOMAC	23	25.43	21	21.99	0	71	6	21	36.00

### Correlation between alignment and clinical outcome

There was no significant correlation between the preoperative mechanical axis and the postoperative WOMAC score. Similarly, there was no significant correlation between the postoperative mechanical axis and the WOMAC score, nor was there a significant correlation between the axis difference and the WOMAC score. The results of the correlation analysis are provided in Table 4.

**Table 4:** Spearman correlations between mechanical alignment and WOMAC; *p < 0.05. **p < 0.01. ***p < 0.001

Spearman-Rho	MecAx Pre	MecAx Post	MecAx Diff	Pain mean	Stiffness mean	Daily activities mean	Total WOMAC mean
MecAx Pre	1.00	0.42*	− 0.56**	− 0.26	− 0.18	− 0.03	− 0.15
MecAx Post	0.42*	1.00	0.41	− 0.34	− 0.25	− 0.23	− 0.33
MecAx Diff	− 0.56**	0.41	1.00	− 0.08	− 0.06	− 0.15	− 0.17
Pain mean	− 0.26	− 0.34	− 0.08	1.00	0.49*	0.86**	0.91**
Stiffness mean	− 0.18	− 0.25	− 0.06	0.49*	1.00	0.57**	0.67**
Daily activities mean	− 0.03	− 0.23	− 0.15	0.86**	0.57**	1.00	0.95**
Total WOMAC mean	− 0.15	− 0.33	− 0.17	0.91**	0.67**	0.95**	1.00

### Correlation between BTU and clinical outcome

**Fig. 5:**
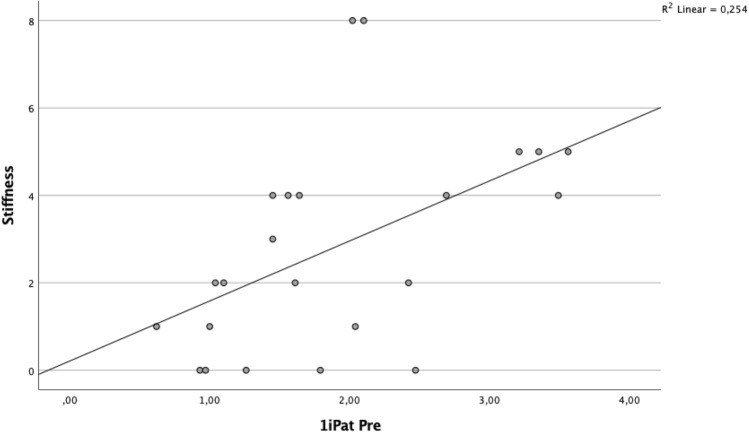
Relationship between WOMAC stiffness score and BTU in area 1iPat

**Fig. 6:**
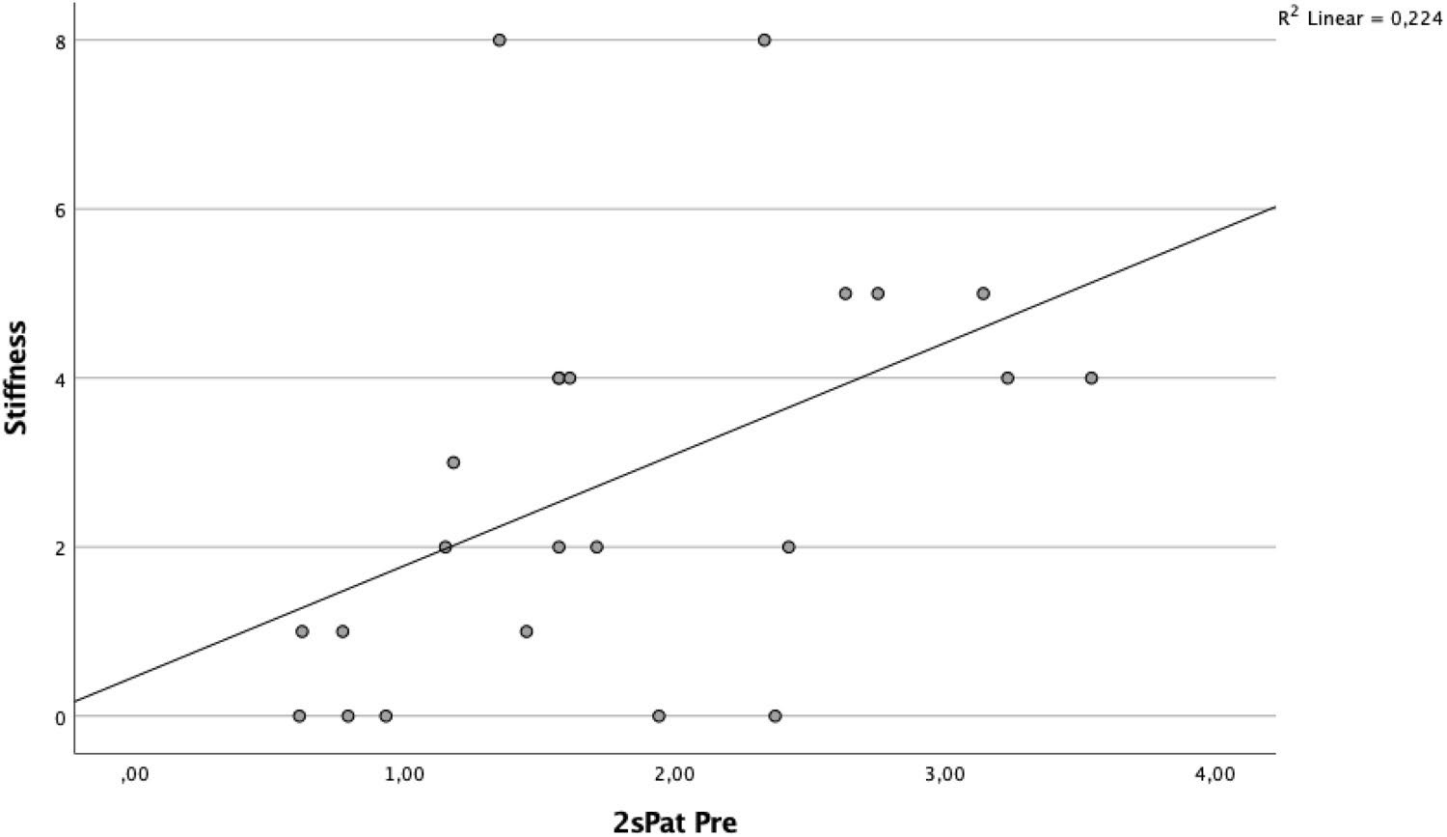
Relationship between WOMAC stiffness score and BTU in area 2sPat

**Fig. 7:**
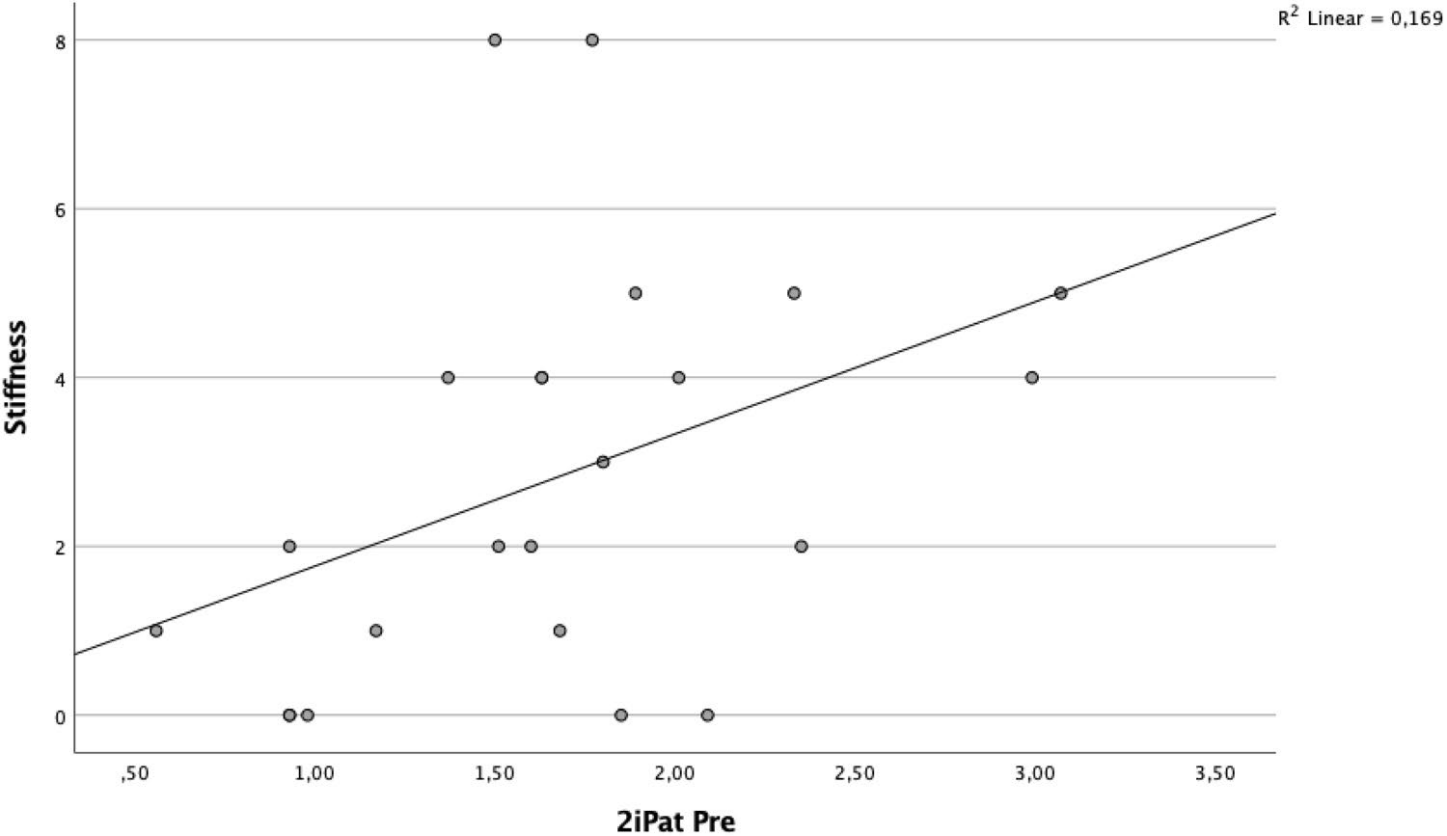
Relationship between WOMAC stiffness score and BTU in area 2iPat

A significant correlation was found between BTU in the patellar region and clinical outcome (Table 5, Figs. 2 and 3). Specifically, the superior lateral area of the patella (1sPat) and the different subcategories of the WOMAC, pain rho = 0.43 (*p* = 0.04, Fig. 5), stiffness rho = 0.59 (*p* = 0.003, Fig. 6) and the total WOMAC score rho = 0.43 (*p* = 0.042, Fig. 4) correlated. The inferior lateral area of the patella (1iPat) correlated with stiffness rho = 0.58 (*p* = 0.004, Fig. 7). The superior medial area of the patella (2sPat) correlated with stiffness rho = 0.54 (*p* = 0.008). The inferior medial area of the patella (2iPat) correlated with stiffness rho = 0.43 (*p* = 0.041).

**Table 5:** Spearman correlations between BTU, alignment and WOMAC score; *p < 0.05. **p < 0.01. ***p < 0.001

Spearman-Rho	MecAx Pre	MecAx Post	MecAx Diff	Pain	Stiffness	Daily activities	Total WOMAC
1saFe Pre	− 0.08	0.34	0.42*	0.00	0.45*	0.04	0.09
2saFe Pre	− 0.09	0.30	0.41	− 0.31	− 0.13	− 0.24	− 0.30
1spFe Pre	0.13	0.42*	0.11	− 0.05	0.16	0.03	0.00
2spFe Pre	0.12	0.35	0.18	− 0.11	0.14	− 0.05	− 0.04
1iaFe Pre	0.28	0.39	0.07	0.05	0.09	− 0.02	0.00
2iaFe Pre	0.01	0.14	0.18	− 0.32	− 0.23	− 0.31	− 0.37
1ipFe Pre	0.24	0.54**	0.15	0.05	0.13	0.09	0.08
2ipFe Pre	0.04	0.09	0.06	− 0.33	− 0.12	− 0.40	− 0.37
1sPat Pre	− 0.31	0.05	0.31	0.43*	0.59**	0.38	0.43*
2sPat Pre	− 0.31	− 0.03	0.23	0.26	0.54**	0.24	0.29
1iPat Pre	− 0.20	0.02	0.24	0.26	0.58**	0.25	0.31
2iPat Pre	− 0.28	0.02	0.34	0.09	0.43*	0.08	0.14
1saTib Pre	− 0.11	0.18	0.34	0.22	0.16	0.06	0.09
3saTib Pre	− 0.10	0.29	0.36	0.12	0.14	0.02	0.11
2saTib Pre	− 0.09	0.25	0.29	− 0.10	− 0.13	− 0.19	− 0.18
1spTib Pre	− 0.10	0.30	0.33	0.11	0.07	0.02	0.02
3spTib Pre	− 0.04	0.18	0.29	0.17	− 0.10	0.10	0.12
2spTib Pre	0.23	0.38	0.12	− 0.30	− 0.16	− 0.33	− 0.30
1iaTib Pre	− 0.09	0.26	0.26	0.29	0.33	0.37	0.35
3iaTib Pre	− 0.07	0.37	0.37	0.18	0.29	0.23	0.22
2iaTib Pre	− 0.05	0.58**	0.49*	0.02	− 0.12	− 0.03	− 0.03
1ipTib Pre	− 0.04	0.17	0.21	0.05	− 0.02	− 0.05	− 0.08
3ipTib Pre	− .02	0.41	0.39	0.14	− 0.13	0.02	0.02
2ipTib Pre	0.21	0.42*	0.22	− 0.18	− 0.18	− 0.17	− 0.20
4Tib Pre	0.06	0.65**	0.49*	− 0.03	− 0.15	− 0.04	− 0.13

### Correlation between demographics and clinical outcome

Comparisons between age, gender and WOMAC did not show a significant correlation (Tables 6, 7).

**Table 6:** Eta-correlations between sex and WOMAC

	*r* =	*p* =
Total WOMAC mean/sex	0.12	ns
Daily activities mean/sex	0.17	ns
Pain mean/sex	0.12	ns
Stiffness mean/sex	0.08	ns

**Table 7:** Spearman correlations between age and WOMAC

			Pain mean	Stiffness mean	Daily activities mean	Total Womac mean
Spearman-Rho	Age OP	rho =	− 0.09	0.13	− 0.02	− 0.03
		Two- tailed *p* value	ns	ns	ns	ns

## Discussion

The most important findings of this study were the following three:

Firstly, preoperative elevated BTU values in the patellar region correlated significantly with a worse clinical outcome. It was previously shown that MOWHTO leads to an increase of the contact pressure in the patellofemoral joint (PFJ) [15, 19, 20, 25, 37, 39]. Kim et al. [19] suggested that the increased loading of the PFJ may be the cause of postoperative pain and be associated with the development and progression of OA in the PFJ. As a matter of fact, several studies were able to show that patellofemoral OA in knees after MOWHTO is progressive on both radiological imaging and knee arthroscopy [4, 24, 41]. Song et al. [36] reported in a study that patients with underlying patellofemoral cartilage degeneration at the time of HTO were particularly affected by the progression of cartilage defects and had a worse clinical outcome compared to patients without such pre-existing cartilage degeneration. A rho of 0.43 can be interpreted as a moderate correlation and leads to a determination coefficient of 0.18. Thus, assuming a causal relationship, the present study shows that increased preload in the superior lateral region of the patella could explain up to 18% of the variance in pain. However, it should be noted that the correlations calculated do not prove a causal relationship between the BTU and WOMAC score [32]. Moreover, as the WOMAC was not collected preoperatively but only postoperatively, it is not clear whether the worse clinical outcome is related to the surgery or not. It cannot be ruled out that the postoperative pain and resulting functional limitations are the normal consequence of progression of pre-existing patellofemoral OA and have not been negatively affected by MOWHTO. However, considering the results of this study in the light of the current literature, it can be assumed that patients with an increased preload in the PFJ are at greater risk for a worse outcome and that the indication for MOWHTO should not be established in patients with an increased load pattern in the PFJ or even osteoarthritis in the PFJ.

Secondly, the hypothesis that clinical outcome is related to alignment correction could not be confirmed. This contradicts the findings of Hernigou, and others [2, 10], who were able to show that both under- and overcorrection of the limb alignment are associated with an inferior outcome. Moreover, recent studies have found negative effects of overcorrection on the progression of patellofemoral OA, which is also in contrast to the findings of our study [4, 24, 29, 41]. However, the reason for our contradictory results could lie in the rather short follow-up period of 2 years, as the studies mentioned above indicate a deterioration after about 7 years [5, 10].

Thirdly, contrary to the results in the literature, no correlation was found between age and outcome [8]. In contrast to the results of van Raaij et al., male gender had no influence on the clinical outcome [40]. However, the results of the present study are only of limited significance due to the small sample size.

Regarding the results of the present study, several limitations must be considered when interpreting the data. In particular, the small number of patients could be a reason for the lack of correlation between preloading of the lateral compartment, the preoperative mechanical axis and the clinical outcome. The second major limitation is that the clinical outcome was only assessed once postoperatively and not preoperatively. Therefore, the clinical outcome of the subjects can only be compared with each other to a limited extent. Moreover, important confounding factors such as patient weight and weight change over time were not recorded, although it has been shown that obese patients with deformities are disproportionately affected by the development and progression of OA [3]. For future studies, it would be advisable to address these issues. It is also known that patient satisfaction and clinical outcome change over time and certain complications occur only after an interval of several years [5, 6, 10]. Therefore, the follow-up time of 24 months in the present study can be considered as questionably short and follow-up studies are advised to consider a longer follow-up period. Moreover, an interesting aspect for future studies would be to see how many of the patients who underwent MOWHTO had a conversion to TKA during follow-up, and to evaluate the preoperative SPECT/CT in these patients for specific loading pattern.

Thus, according to the results of the present study, conducting a preoperative SPECT/CT scan may help the treating surgeons in their day-to-day work to decide which patients would be better recommended for an alternative treatment method due to the risk of a worse clinical outcome. However, as promising as the possible use of SPECT/CT as a predictive tool seems, it needs to be investigated in further studies.

## Conclusion

This study highlights the role of preoperative SPECT in modern knee surgery to obtain information about the loading pattern on different compartments of the knee. Despite its limitations, this study could help surgeons on a routine basis by identifying patients in whom special care is required. In patients with increased BTU in the patellar region, the indication for MOWHTO should be made with all due caution and possible alternative treatment options should be evaluated to avoid dissatisfied patients.
